# A brief history of artificial intelligence embryo selection: from black-box to glass-box

**DOI:** 10.1093/humrep/dead254

**Published:** 2023-12-07

**Authors:** Tammy Lee, Jay Natalwala, Vincent Chapple, Yanhe Liu

**Affiliations:** Fertility North, Joondalup Private Hospital, Joondalup, Western Australia, Australia; School of Human Sciences, University of Western Australia, Crawley, Western Australia, Australia; Fertility North, Joondalup Private Hospital, Joondalup, Western Australia, Australia; Fertility North, Joondalup Private Hospital, Joondalup, Western Australia, Australia; Fertility North, Joondalup Private Hospital, Joondalup, Western Australia, Australia; School of Human Sciences, University of Western Australia, Crawley, Western Australia, Australia; Faculty of Health Sciences and Medicine, Bond University, Robina, Queensland, Australia; School of Medical and Health Sciences, Edith Cowan University, Joondalup, Western Australia, Australia

**Keywords:** embryo selection, time-lapse videography, artificial intelligence, black-box, glass-box, interpretability, subjectivity, machine learning, deep learning, explainability

## Abstract

With the exponential growth of computing power and accumulation of embryo image data in recent years, artificial intelligence (AI) is starting to be utilized in embryo selection in IVF. Amongst different AI technologies, machine learning (ML) has the potential to reduce operator-related subjectivity in embryo selection while saving labor time on this task. However, as modern deep learning (DL) techniques, a subcategory of ML, are increasingly used, its integrated black-box attracts growing concern owing to the well-recognized issues regarding lack of interpretability. Currently, there is a lack of randomized controlled trials to confirm the effectiveness of such black-box models. Recently, emerging evidence has shown underperformance of black-box models compared to the more interpretable traditional ML models in embryo selection. Meanwhile, glass-box AI, such as interpretable ML, is being increasingly promoted across a wide range of fields and is supported by its ethical advantages and technical feasibility. In this review, we propose a novel classification system for traditional and AI-driven systems from an embryology standpoint, defining different morphology-based selection approaches with an emphasis on subjectivity, explainability, and interpretability.

## Graphical Abstract

**Graphical Abstract:**
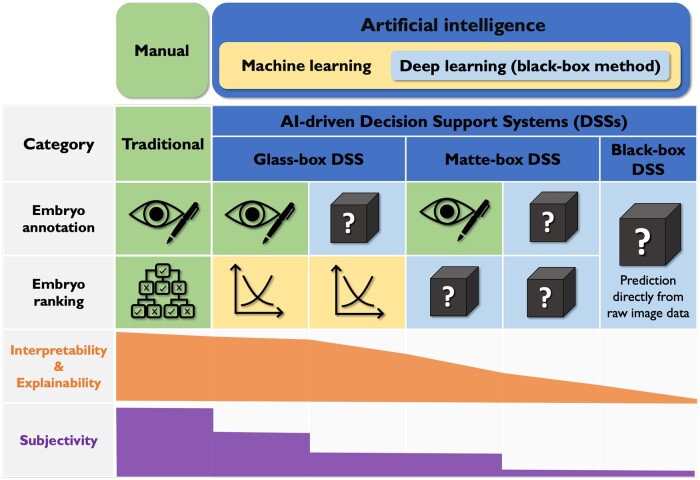
A proposed classification system for artificial intelligence embryo selection models with different subjectivity, interpretability, and explainability.

## Introduction

Optimum embryo selection is critical in assisting patients undergoing IVF treatment to achieve pregnancy in the least number of transfers possible (Gardner *et al.*, 2015). However, a well-recognized challenge in morphology-based embryo selection is inter- and intra-operator subjectivity (Sundvall *et al.*, 2013; Storr *et al.*, 2017; Bormann *et al.*, 2020). The clinical application of time-lapse videography (TLV) in IVF enables identification of novel biomarkers for embryo selection, with potentially enhanced performance when coupled with artificial intelligence (AI) (Liu *et al.*, 2020a; Riegler *et al.*, 2021). Machine learning (ML) is a subgroup of AI offering the potential to minimize operator-associated subjectivity and improve embryo selection. In the past few years, there has been a boom in the clinical application of deep learning (DL) in embryo selection (Curchoe and Bormann, 2019). DL is a subgroup of ML and is predominantly based on artificial neural networks with multiple hidden layers. These hidden layers, however, make its reasoning process uninterpretable, giving rise to an alternative term for DL—the black-box (Tu, 1996). As a result, this lack of transparency attracts growing ethical and societal concerns from IVF professionals (Afnan *et al.*, 2021).

**Figure 1.**
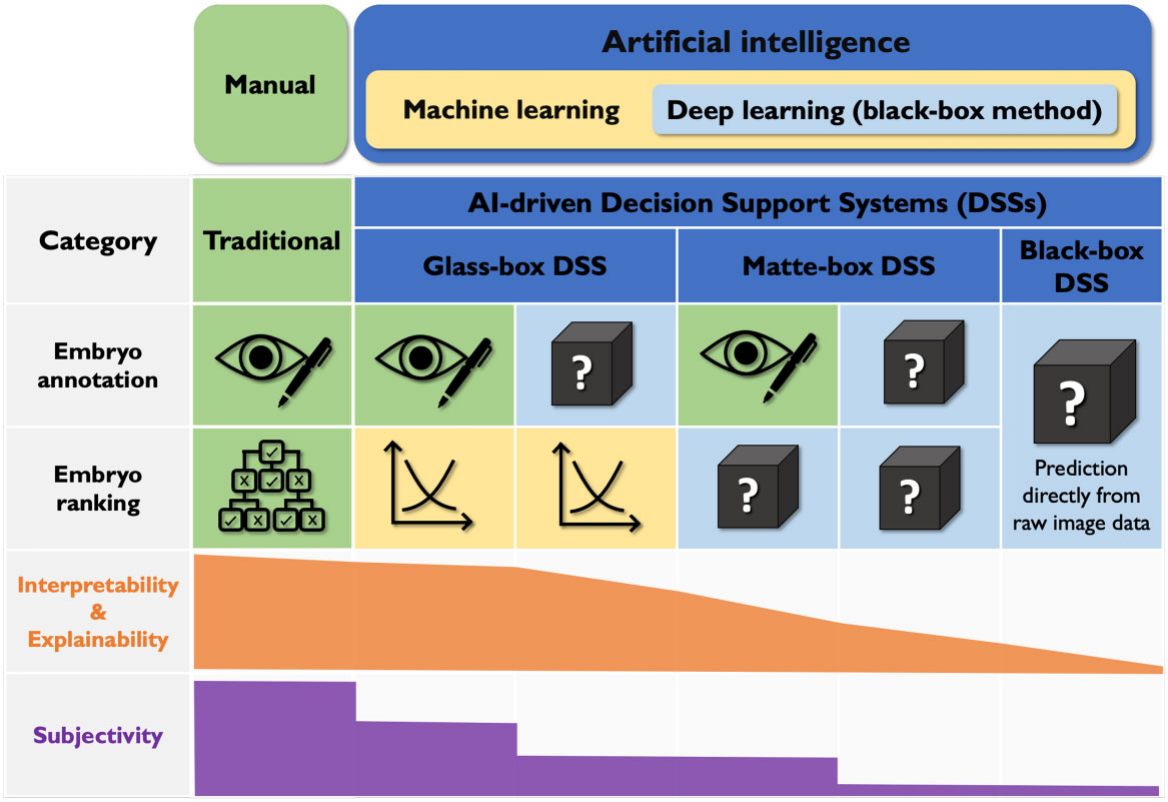
A proposed classification system for artificial intelligence embryo selection models with different subjectivity, interpretability, and explainability.

The pursuit for AI-driven applications in modern human society is not dissimilar from the old day’s ‘gold rush’ and is fraught with risks. With the effectiveness of most AI-driven decision support systems (DSSs) in embryo selection yet to be validated via randomized controlled trials (RCTs), the sacrifice of interpretability cannot be justified (Afnan *et al.*, 2021). A recent methodology comparison of 12 algorithms developed for blastocyst viability prediction revealed that logistic regression, as an interpretable ML method, outperformed the other 11 ML methods, including its black-box counterparts (Bamford *et al.*, 2023). This is a timely reminder for IVF professionals to perhaps decelerate from the black-box hype and consider alternative AI-driven DSSs for embryo selection, so that proper guardrails can be established under a stronger regulatory framework. To facilitate appropriate differentiation of AI decision tools, this mini-review aims to propose a novel classification system for traditional and AI-driven DSSs for morphology-based embryo selection, by considering each category’s embryo annotation and ranking steps. AI-driven DSSs are further classified into black-box, glass-box, and matte-box subgroups with a focus on their overall interpretability, explainability, and subjectivity (Fig. 1).

## Traditional embryo selection

### The two steps in traditional embryo selection

Historically, traditional embryo selection utilizes two distinct sequential steps, namely annotation of the raw image(s) of the embryo, followed by ranking amongst a cohort of embryos. Accurate annotation enables extraction of biologically meaningful variables, either qualitative or quantitative (Liu *et al.*, 2020a). Annotation can be derived from both static images via microscopic observations at defined times during embryo culture (Alpha Scientists in Reproductive Medicine and ESHRE Special Interest Group of Embryology, 2011) and morphokinetic data (i.e. TLV-based images captured over time in culture, which can be manually generated). However, static assessment does not allow annotation of quantitative TLV parameters such as time of the first cell cleavage to a two-cell embryo, duration of cell cycles, and start of blastulation (Ciray *et al.*, 2014). Subsequently, a ranking model is built by incorporating all contributing variables through reasoning, supported by either in-house studies or evidence in the literature (Alpha Scientists in Reproductive Medicine and ESHRE Special Interest Group of Embryology, 2011; Meseguer *et al.*, 2011). In traditional embryo selection, subjectivity issues are expected to exist in both the annotation (the assessing embryologist’s identification of developmental events and/or grading of specific morphological features) and ranking steps (prognostic weightings amongst such features that the assessing embryologist can see), despite its high interpretability (Fig. 1: ‘Traditional’ column).

### Traditional embryo selection using static images

Morphology-based embryo selection with standard incubation has historically been the predominant method of traditional embryo selection. In cleavage-stage embryos, cell number, level of fragmentation and the presence of multi-nucleation can be assessed (Alpha Scientists in Reproductive Medicine and ESHRE Special Interest Group of Embryology, 2011), and are all well-recognized to be highly subjective considering the quality of recording from a static observation and operator subjectivity. The Gardner blastocyst grading system is a popular traditional approach and combines extended culture and static morphological grading of expansion stage, inner cell mass (ICM), and trophectoderm (TE) (Gardner and Schoolcraft, 1999). Moderate to high levels of inter- and intra-operator consistencies are observed when using this system (Sundvall *et al.*, 2013; Storr *et al.*, 2017).

Although the accuracy of pregnancy prediction is lower in traditional morphology-based embryo selection compared to AI-driven DSSs (VerMilyea *et al.*, 2020; Chavez-Badiola *et al.*, 2020a; Loewke *et al.*, 2022), it is a globally well-established protocol that involves minimum investment and training for new embryologists. Development of advanced models (i.e. AI-driven DSSs as discussed in below sections) may require intellectual statistical input and/or capital investment if commercial third parties are adopted. Therefore, many clinics may opt to persist with morphology-only based methods of embryo selection.

### Traditional embryo selection using TLV images

The clinical introduction of TLV offers potentially decreased inter-operator subjectivity in the annotation step in comparison to static observation (Scott *et al.*, 2021), while also enabling identification of novel viability biomarkers (Liu *et al.*, 2020b; Bickendorf *et al.*, 2023). Compared to static morphology, the subjectivity of the ranking step in traditional embryo selection using TLV can be further reduced by use of an algorithm with a defined set of rules to determine the most viable embryo. Different approaches to achieve this include the incorporation of manually annotated embryonic features to generate simple manual decision trees (Meseguer *et al.*, 2011) (Fig. 1: ‘Traditional’ column). However, the subjective nature of manual annotation and the limited number of viability biomarkers available in this category form a bottleneck for further improvement in embryo selection. AI-driven DSSs have the potential to leverage such challenges through reduced subjectivity and ability to assist in defining more biomarkers. Furthermore, AI-driven DSSs also have the potential to detect discrete or novel variables that are otherwise undetectable by the human eye.

## AI-driven DSSs

In modern AI methodology, an important distinction to make is that DL or black-box methods are a subcategory of ML, which falls within the broader umbrella of AI (Fig. 1). In the field of embryo selection, the majority of black-box methods utilize neural networks such as convolutional neural networks (Dimitriadis *et al.*, 2022). Black-box DSSs, as defined in this article, receive raw imaging data as input, often in video form, to directly predict pregnancy without defining any embryonic features (cleavage timings, abnormal cleavage, etc.) (Fig. 1: ‘Black-box DSS’ column). In addition to black-box DSSs (one-step process), black-box methods can also be included in two-step DSSs by automatically annotating known embryonic features (such as morphokinetics or morphology, rather than directly predicting pregnancy) in the first step of the two-step process; and/or ranking embryos using annotated embryonic features in the second step. Therefore, glass-box and matte-box DSSs also involve black-box methods in one or both steps of their two-step process while still enabling sense checking to some extent. To elaborate on two-step AI-driven DSSs, a matte-box DSS with automatic annotation of embryonic features can combine a black-box first step and a ranking second step that also uses black-box methods (matte-box with automatic annotation, Fig. 1). Imaging data can also be manually annotated for embryonic features first, followed by a ranking step using black-box methods to predict pregnancy potential (matte-box DSS with manual annotation). Embryo ranking black-box methods contrast with more interpretable ML methods, which have clear constraints and allow an understanding of how a model reached its output. Glass-box DSSs have an embryo ranking step conducted by interpretable ML methods and can receive either manually annotated (glass-box DSS with manual annotation) or automatically annotated embryonic features (glass-box DSS with automatic annotation). Interpretable ML methods (those that do not involve black-box methods in the ranking step) are mostly statistics oriented, such as logistic regression, decision trees, random forest, and Bayesian networks.

### Black-box DSS and its associated issues

With the rising hopes for, and accelerating popularity of, AI applications in embryo selection (Curchoe and Bormann, 2019), black-box DSSs have dominated recent literature in embryo selection (Dimitriadis *et al.*, 2022). The recent application of many black-box DSSs has merged the two-step embryo selection process and hence removed its inherited interpretability and explainability. Interpretability can be defined as ‘the ability to explain or present in understandable terms to a human’ (Doshi-Velez and Kim, 2017) or ‘the degree to which a human can understand the cause of a decision’ (Miller, 2019). In other words, the more interpretable a model is, the easier it is to identify cause-and-effect relationships within its inputs and outputs (Linardatos *et al.*, 2020). For example, an ML model built on linear regression is interpretable where the trained model consists of an equation with positive or negative coefficients multiplying the values of relevant covariates. Although it is often used interchangeably with interpretability, explainability has been defined as ‘the collection of features of the interpretable domain, that have contributed for a given example to produce a decision’ (Montavon *et al.*, 2018). For example, a black-box model is considered explainable if the importance of the variables used as input can be quantified.

One influential black-box DSS was reported by Tran *et al.* (2019), showing a near perfect prediction (AUC >0.9) of fetal heart detection by raw TLV data as input. However, its unbalanced dataset (80% of the included embryos were not transferred) and the metrics used to measure performance were subsequently criticized by different groups (Kan-Tor *et al.*, 2020; Chavez-Badiola *et al.*, 2020b). The authors, however, have rightly pointed out in the ‘Discussion’ section that the poor understanding of the algorithm’s decision-making logic required further investigation (Tran *et al.*, 2019). Different approaches were also reported to predict embryo viability using static blastocyst images captured by light microscopy or different TLV devices (VerMilyea *et al.*, 2020; Diakiw *et al.*, 2022). However, if a long-term bias were to occur on the embryos selected via black-box DSS, it would be difficult to detect under such a black-box setup in the short term before it is perpetuated and further amplified over time, especially when a surrogate short-time end point, such as ploidy or fetal heart detection (rather than live birth or healthy birth), had been used for training (Afnan *et al.*, 2021). For example, reports have shown sex-linked morphokinetic and morphometric differences in human zygotes/embryos (Bronet *et al.*, 2015; Orevich *et al.*, 2022; Kilbee *et al.*, 2023). In populations where individuals are selected by algorithms involving such features, this could lead to downstream effects. Indeed, while a high-quality large dataset is recognized as an important determinant of performance and robustness in black-box DSSs (Bormann *et al.*, 2020; Curchoe *et al.*, 2020; Hickman *et al.*, 2020), more interpretable or explainable algorithms would have the intrinsic advantage of minimizing such risks by the early detection/correction of any foreseeable bias, as reasoning in the algorithm’s decisions can be pinpointed (Rudin, 2019). Therefore, considering the important role that IVF plays in human reproduction, we emphasize that embryo selection should be highly interpretable and explainable.

From a subjectivity standpoint, black-box AI embryo selection is advantageous over traditional methods (Fig. 1: ‘Black-box DSS’ column). Fordham *et al.* (2022) recently presented inferior reproducibility in blastocyst ranking by embryologists in reference to a purpose-built deep neural network model. Furthermore, a study by Loewke *et al.* (2022) reported a 5–12% improvement in clinical pregnancy rate via a convolutional neural network model compared to embryo selection by manual blastocyst ranking. Interestingly, the authors reported that the use of a low-quality microscope in one of the 11 participating clinics had failed to achieve optimization in clinical pregnancy rate, highlighting the importance to consider systematic factors on top of embryonic data (Loewke *et al.*, 2022). Nevertheless, it is often challenging to label all known confounders during the training process of a black-box algorithm, let alone the unknown confounders. This further highlights the critical role that RCTs play in effective evaluation of any new intervention before routine clinical application.

Another important issue is that of transferability (Meseguer and Valera, 2021), where an algorithm that is trained under specific clinical settings does not perform well when applied to a different clinical environment (Petersen *et al.*, 2016; Barrie *et al.*, 2017; Liu *et al.*, 2019; Johansen *et al.*, 2023). An external algorithm trained elsewhere should only be introduced clinically following thorough in-house validation as different factors can affect embryo morphokinetic profiles and endometrial receptivity. Potential contributors to the transferability issue include: impacts of culture conditions (Zaninovic *et al.*, 2013), culture media (van Duijn *et al.*, 2022), patient population (Freour *et al.*, 2013), and controlled ovarian stimulation regimes (Munoz *et al.*, 2013). Commercial algorithms may benefit from access to larger datasets so that a more balanced transferability/performance can be achieved, though large high-quality datasets are difficult to obtain (Hickman *et al.*, 2020). There are, however, alternative paths to address the transferability issue by utilizing more transferable qualitative TLV measures, such as abnormal cleavage (Liu *et al.*, 2020a) or spontaneous blastocyst collapse (Bickendorf *et al.*, 2023). Annotation for embryo abnormal cleavage patterns is still mostly performed manually by an embryologist, while newer reports, such as on blastocyst spontaneous collapse (Cimadomo *et al.*, 2022), are starting to engage AI-powered tools to enable automated annotation. In fact, automated annotation is worth further attention considering its important role in both improvement of consistency and accuracy in annotating time-lapse images and maintaining better interpretability of embryo selection. This could be achieved by ensuring the two-step process is maintained as demonstrated in the matte-box and glass-box DSSs promoted in this article.

### Matte-box DSSs (manual or automatic annotation followed by a black-box ranking step)

A key element of a matte-box DSS in embryo selection is the clear separation of the annotation and ranking steps; annotation is completed manually or automatically, followed by a black-box ranking step (Fig. 1: ‘Matte-box DSS’ columns). Such clear separation of the two steps in a matte-box DSS is preferrable over black-box DSSs owing to: its increased interpretability by involving defined embryonic features; availability of sense checking by users on the annotations; and the possibility of applying confidence limits. Using manual annotation, Bori *et al.* (2020) incorporated a dozen morphokinetic parameters, both conventional and novel, into an artificial neural network to predict clinical pregnancy. However, reports in the matte-box DSS category using combined automated annotation and black-box ranking are currently limited. There are claims that certain software is able to annotate a range of TLV measures but embryologist supervision/correction is often required. Therefore, a dividing line between manual and automated annotation would be unclear in such cases.

Regarding automated annotation independently, the automated segmentation of areas of interest in an embryo image has been one of the most studied areas, with the potential to reduce operator-related subjectivity. These include the automated segmentation of the ICM (Kheradmand *et al.*, 2017; Rad *et al.*, 2017), TE (Rad *et al.*, 2020), zona pellucida thickness (Yee *et al.*, 2013; Rad *et al.*, 2018) or a combination of the three in a blastocyst (Filho *et al.*, 2012; Farias *et al.*, 2023). The use of TLV further boosted advancements in automated detection of pronuclei (Fukunaga *et al.*, 2020) and subsequent embryo cleavage stages (Dirvanauskas *et al.*, 2019; Raudonis *et al.*, 2019). An important milestone study was reported by Feyeux *et al.* (2020), which automated the annotation of morphokinetic parameters ranging from early cleavage stages to the expanded blastocyst stage. This progress, however, has purely focused on the annotation aspect without involving any ranking step. More recently, Cimadomo *et al.* (2022) investigated blastocyst collapsing events in much detail by annotating start/end times of blastocyst collapse as well as the degree of blastocoel shrinkage. This study underlined blastocyst collapsing as a potential viability marker. However, the automated detection of abnormal cleavage events has progressed relatively slowly. Reverse cleavage is among these, which requires close tracking of both karyokinesis and cytokinesis activities (Liu *et al.*, 2014). Furthermore, research in this field would expect heavy involvement of embryologist(s) to facilitate the data labeling process during training for such algorithms. Ground truth (i.e. embryologists’ consensus) used in automatic annotation studies is far from objective in comparison to fetal heart detection or live birth, which are mostly used. While automatic annotation can potentially reduce subjectivity, human associated confounders could still be rampant and thus have downstream effects in embryo ranking.

### Glass-box DSSs (manual or automatic annotation followed by interpretable ML in the ranking step)

Image(s), static or TLV, in the glass-box DSS category can be manually or automatically evaluated, followed by a ranking step via interpretable ML methods such as Bayesian networks, ML decision trees or multi-variate logistic regression (Fig. 1: ‘Glass-box DSS’ columns). For the embryo ranking step, ML methods outside of DL are more interpretable, as they enable clear demonstration of variables involved and the weighting information of each of them. Bayesian networks, as a probabilistic graphical model, allow coherent inference by enabling calculation of probabilities for known variables in the network and have been used in embryo selection (Morales *et al.*, 2008; Hernandez-Gonzalez *et al.*, 2018). With a large multi-center dataset, Petersen *et al.* (2016) used an ML decision tree to rank embryos into five groups depending on their implantation potential. Decision trees are considered interpretable and explainable because of their tree-like structure and the way their decision rules provide insights into how the model reached its output.

Moreover, multi-variate logistic regressions also allow relative weightings of coefficients for a given outcome. As mentioned previously, the Gardner blastocyst grading system is considered a traditional embryo selection method. However, varying blastocyst observation timings between laboratories proved to be challenging to control when linking static morphology features to treatment outcomes, considering the dynamic nature of embryo development (Liu *et al.*, 2022). Using a multi-variate logistic regression and incorporating calculated weightings for each contributing variable, the same study presented a numerical scoring system that could potentially minimize operator-related subjectivity at the ranking step (Liu *et al.*, 2022). In the study, four variables were input into the logistic regression and coefficients were used as weightings for each of the variables. Therefore, owing to the calculated weightings, the model is interpretable and can result in less disagreement between embryologists. More recently, following manual morphokinetic annotation, Bamford *et al.* (2023) established that a logistic regression model was superior amongst 12 different models in blastocyst ploidy prediction. The other 11 models included black-box DSSs, reinforcing the fact that further performance comparisons amongst matte-box, glass-box, and black-box DSSs are warranted.

By incorporating automatically annotated features with minimized operator-related subjectivity, glass-box DSSs have the potential to achieve optimized interpretability and robustness. Using TLV, Khosravi *et al.* (2019) utilized deep neural networks to automate blastocyst classification against five experienced embryologists, which resulted in near perfect performance (AUC >0.98). In this study, an embryologist majority voting procedure (blastocyst quality determined by agreement from at least three of the five embryologists) was used to classify blastocyst images (Khosravi *et al.*, 2019). This was followed by an ML decision tree to predict live birth.

A glass-box DSS was recently reported based on a day 3 embryo dataset, involving only two automatically annotated embryo morphokinetic measures, namely the durations of two- and three-cell stages (Valera *et al.*, 2023). These measures were subsequently input into an ML decision-tree-based ranking model giving rise to five categories. This process could be replicated once all the parameters are automatically annotated. Future glass-box DSSs involving an expanded panel of automatically annotated embryonic parameters and interpretable ML models are warranted in the coming years, owing to their relative trade-off of interpretability, explainability, and subjectivity in comparison to other approaches.

### Alternative paths to enhanced interpretability and explainability

Recently, attempts to understand how black-box DSSs arrive at their output from embryo imaging data have been made by different groups (Enatsu *et al.*, 2022) and this is a way to potentially improve explainability (Curchoe *et al.*, 2020). Studies have used the gradient-weighted class activation method (Enatsu *et al.*, 2022; Loewke *et al.*, 2022) or attribution algorithms, which include integrated gradients and occlusion maps (Loewke *et al.*, 2022). Both techniques enable visualization of areas on the embryo that contribute most to the predicted outcome. Although these studies do not lead to complete interpretability because of their black-box nature, they provide important insights for future studies, especially when exploring novel biomarkers for embryo viability. Nevertheless, the absence of a clear border between the annotation and ranking steps hampers its development toward further interpretability.

Combining clinical parameters with embryo morphological data to train an algorithm is another way toward improved explainability. This is because such AI-driven DSSs combine a black-box DSS (one-step process) to analyze the embryos, and a more interpretable ML model to consider clinical values of the patient, to output the final embryo ranking. Enatsu *et al.* (2022) reported a hybrid AI-driven DSS, which combined static blastocyst image data and clinical parameters including female age, pregnancy history, hormonal levels, etc. The hybrid model resulted in a 4.5% AUC rise in prediction of clinical pregnancy compared to image data alone. Similarly, by using a black-box method and TLV data, Duval *et al.* (2023) demonstrated superior predicting performance in its hybrid model. Clinical pregnancy prediction was improved by further incorporating clinical parameters such as oocyte age, total gonadotrophin dose intake, number of embryos generated and endometrial thickness. Although the addition of meaningful clinical parameters in these algorithms elevates their overall interpretability and explainability, the embryonic elements in these models remain uninterpretable. Hybrid models highlight that the embryo alone may not be the best predictor of treatment outcome and the confounding impacts of clinical factors should also be considered.

## Current challenges and future perspectives

In this article, we reviewed different embryo selection approaches from an embryology perspective. We included examples of published studies (Supplementary Table S1) to assist in defining each category and to differentiate between studies. There was, however, a skewed distribution of available studies in each category with the matte-box approaches being the least available. This is unfortunately not apparent in our table as we did not perform a systematic review. Based on our findings, we call for more research in the following areas: development of more glass-box DSSs to increase the interpretability of AI-driven DSSs in general; more evidence in performance evaluation between different methodologies (including approaches such as logistic regression); comprehensive performance metrics analyses when measuring algorithm performance, as proposed by Riegler *et al.* (2021); automation in TLV parameter annotation (preferably qualitative TLV measures for better transferability between laboratories) coupled with advanced ranking methods; RCTs to provide robust validation of any new algorithm; and creation of a publicly available open repository of a diverse, high-quality large embryo image dataset for algorithm development, validation and benchmarking, for improved standardization and robustness.

## Conclusion

Traditional embryo selection involves two steps, namely embryo annotation (static image or morphokinetics) and ranking amongst a cohort of embryos, and is therefore highly interpretable although their intrinsic subjective nature hampers standardization. In black-box DSSs, the two-step border is absent. In glass-box and matte-box DSSs, the annotation step can either be manually completed by a human or automatically executed by black-box methods. Secondly, glass-box and matte-box DSSs use more interpretable ML and black-box methods, respectively, for embryo ranking. The performance advantage of black-box DSSs has recently been challenged and glass-box DSSs are more interpretable than black-box DSSs owing to the use of interpretable ML methods in the ranking step. Successful implementation of glass-box DSSs will require close interdisciplinary collaboration, where embryologists play a vital role not only in identifying more biologically meaningful embryonic features but also in assisting in the training of automatic annotation of these features. An increasing awareness of black-box issues amongst IVF professionals, patients, and software and device developers, as well as regulatory bodies, would foster a shift to more interpretable and explainable glass-box DSSs.

## Supplementary Material

dead254_Supplementary_Table_S1

## Data Availability

All data generated in this study are included in the full text and Supplementary Table S1.
