# Supplementary material for: A brief history of artificial intelligence embryo selection: from black-box to glass-box
Source: Hum Reprod. 2023 Dec 7;39(2):285–92. doi: 10.1093/humrep/dead254 (PMC11016335; doi:10.1093/humrep/dead254)
Supplement: dead254_Supplementary_Table_S1 [file dead254_supplementary_table_s1.pdf]

OXFORD
human reproduction
Human Reproduction, 2023, 1–2
https://doi.org/10.1093/humrep/dead254
Supplementary Data

Supplementary Table S1. Examples of morphology-based embryo decision support systems for traditional and artificial intelligence-based categories.

Study	Category	Input	Method to develop algorithm	Prospective/ retrospective	Number of centers	Sample size	Embryo population	Endpoint(s)	Training/validation/testing	Performance (AUC, accuracy, precision, sensitivity etc.)
Meseguer *et al.* (2011)	Traditional	Six morphokinetic parameters (t2, t3, t4, t5, cc2, s2), uneven blastomere size, multinucleation	Manual decision tree	Retrospective	1	247 embryos	D3, ICSI only	CP	Validation only	AUC 0.72 for TLV, AUC 0.64 for morphology alone
Liu *et al.* (2016)	Traditional	Conventional morphological D3 grade, morphokinetic patterns (t5_pnf, s2) and abnormal cleavage patterns (RC, DC, <6ICCP)	Manual decision tree	Retrospective	1	270 embryos	D3	CP	Prospectively validated with 66 embryos in two different media types	AUC 0.762
Hernandez-Gonzalez *et al.* (2018)	Glass-box - manual annotation	Embryo grade and clinical parameters	Bayesian networks	Retrospective	1	696 embryos from 330 patients	D2, IVF and ICSI	CP of at least one embryo	Two training datasets (one with embryo data only and the other that combined clinical parameters) followed by validation on 253 embryos from 134 cycles	Ranges of different Bayesian classifiers in two testing datasets (one with just embryonic data and the other that combined clinical data)were as follows: accuracy (0.76-0.86), recall (0-0.49), precision (0-0.4), F1 (0-0.36) and PPR (0-0.23)
Morales *et al.* (2008)	Glass-box—manual annotation	(i) Cleavage stage morphology of the three embryos that were implanted and (ii) clinical data	Bayesian networks	Retrospective	1	89 cycles from 63 patients, 189 embryos. Three embryos transferred at a time	D2, IVF and ICSI, fresh and frozen	CP of at least one embryo	Validation only. Leave-one-out cross-validation method	Accuracy of different Bayesian classifiers ranged from 0.63-0.71
Petersen *et al.* (2016)	Glass-box - manual annotation	Morphokinetic parameters (2PN, tPNf, t2, t3, t5, t8)	Decision tree and numerical scoring system	Retrospective	24	Implantation: 3275 embryos. Blast formation: 11 218 embryos cultured to D5	D3 ET, IVF and ICSI	Implantation potential, blastocyst development and blastocyst quality	5-fold cross-validation testing of complete data set	Implantation potential AUC 0.65, predicting blastocyst development on day 3 AUC 0.745, predict blastocyst quality on day 3 AUC 0.679
Liu *et al.* (2022)	Glass-box - manual annotation	Static blastocyst image, female age, embryo developmental stage, embryo morphology, HPI at D5	Multivariate logistic regression	Retrospective	11	4851 embryos	D5 SET	CP and LB	80% training and 20% 5-fold cross-validation	AUC ~0.7
Khosravi *et al.* (2019)	Glass-box - automatic annotation	TLV and female age	CNN to predict blastocyst quality, followed by decision tree to predict LB	Retrospective	1	10 148 embryos to predict blastocyst quality (although not all utilized due to image quality). 2182 embryos with patient age information and pregnancy outcome results	Blastocysts	Predict blastocyst quality, LB	70% training and 30% to validation and testing. Further validation on two external clinics	AUC 0.987 on blind test set, 0.9694 accuracy to predict good-quality and poor-quality embryos, 97.5% accuracy to predict embryo grade
Valera *et al.* (2023)	Glass-box - automatic annotation	TLV: P2 (t3-t2), P3 (t4-t3), oocyte age and number of cells considered	Generalized estimating equations for ranking	Retrospective	1	5027 embryos	D3, ICSI only	Blastocyst development, implantation, LB, euploidy prediction	Validation only	Algorithm excluding blastocyst morphology: AUC 0.61 for implantation, AUC 0.622 for LB

(continued)

Supplementary Table S1. Continued

Study	Category	Input	Method to develop algorithm	Prospective/ retrospective	Number of centers	Sample size	Embryo population	Endpoint(s)	Training/validation/testing	Performance (AUC, accuracy, precision, sensitivity etc.)
Bori *et al.* (2020)	Matte-box - manual annotation	Morphokinetic parameters (distance and speed of pronuclear migration, blastocyst expanded diameter, inner cell mass area, cell cycle length in trophectoderm) and conventional morphokinetic parameters	Multilayer perceptron	Retrospective	1	637 embryos	ICSI only, SET	Prediction of clinical pregnancy	85% training and validation and 15% testing	Ranges for four ANNS were as follows: sensitivity (0.82-0.88), specificity (0.46-0.67), accuracy (0.71-0.76), F score (0.78-0.80) and AUC (0.64-0.77)
Tran *et al.* (2019)	Black-box	TLV	CNN	Retrospective	8	10 638 embryos, 1835 cycles from 1648 individual patients	Blastocysts	Probability of fetal heart pregnancy	80% training and 20% testing	AUC 0.93
Diakiw *et al.* (2022)	Black-box	Static blastocyst image	CNN	Retrospective and prospective	10	5050 embryos	Blastocysts	Ploidy status	Train-validate cycle process. Untrainable data cleansing and distillation training and holdback validation dataset	Sensitivity 74.6%, specificity 48.6%, overall accuracy 65.3%, PPV 72.4%, NPV 51.3%, MCC 0.235, AUC 0.68
VerMilyea *et al.* (2020)	Black-box	Static blastocyst image	Ensemble modelling to combine computer vision methods and CNN (ResNet and DenseNet)	Retrospective	11	8886 embryos	Blastocysts	Prediction of CP	Train-validate cycle process. Pilot study to develop initial model used 5282 images from a single clinic and blind test dataset of 1000 embryos. Model then further developed from an additional 3604 images from 11 clinics and blind test sets were done on 1667 embryos	Sensitivity 0.71, specificity 0.605, overall weighted accuracy >0.63, combined accuracy across both viable and non-viable embryos 0.643
Fordham *et al.* (2022)	Black-box	TLV	DNN	Retrospective	2	136 embryos	Blastocysts, IVF (2%) and ICSI (98%)	Implantation probability grade	10-fold cross-validation. Each iteration: 7-fold training, 2-fold validation, 1-fold testing	Overall accuracy 62.5%, F1 67.1%, sensitivity 80%, NPV 71.7%, PPV 57.8%, AUC 0.7

AMH, anti-Müllerian hormone; CNN, convolutional neural network; CP, clinical pregnancy; D3, day three; D5, day five; D6, day 6; DET, double embryo transfer; DNN, deep neural network; HPI, hours post-insemination; LB, live birth; MCC, Matthew correlation coefficient; MLP, multilayer perceptron; NPV, negative predictive value; PPR, predicted positive rate; PPV, positive predictive value; SET, single embryo transfer; TLV, time lapse videography; RC, reverse cleavage; DC, direct cleavage; ICCP, intercellular contact points; HPI, hours post-insemination.
